# Antenatal Care Strengthening in Jimma, Ethiopia: A Mixed-Method Needs Assessment

**DOI:** 10.1155/2014/945164

**Published:** 2014-08-31

**Authors:** Sarah Fredsted Villadsen, Britt Pinkowski Tersbøl, Dereje Negussie, Abebe GebreMariam, Abebech Tilahun, Henrik Friis, Vibeke Rasch

**Affiliations:** ^1^Department of Nutrition, Exercise and Sports, Faculty of Science, University of Copenhagen, Rolighedsvej 25, 1958 Frederiksberg C, Denmark; ^2^Department of Public Health, Faculty of Health and Medical Sciences, University of Copenhagen, Oester Farimagsgade 5, P.O. Box 2099, 1014 Copenhagen K, Denmark; ^3^Department of International Health, Immunology and Microbiology, Faculty of Health and Medical Sciences, University of Copenhagen, Blegdamsvej 3, 2200 Copenhagen N, Denmark; ^4^Department of Obstetrics and Gynaecology, College of Public Health and Medical Sciences, Jimma University, P.O. Box 378, Jimma, Ethiopia; ^5^Department of Population and Family Health, Jimma University, P.O. Box 378, Jimma, Ethiopia; ^6^JUCAN Research Collaboration, Jimma University, P.O. Box 378, Jimma, Ethiopia; ^7^Department of Obstetrics and Gynaecology, Odense University Hospital, Sdr. Boulevard 29, 5000 Odense C, Denmark

## Abstract

*Objective.* We assessed how health system priorities matched user expectations and what the needs for antenatal care (ANC) strengthening were for improved maternal health in Jimma, Ethiopia. *Methods.* A questionnaire survey among all recent mothers in the study area was conducted to study the content of ANC and to identify the predictors of low ANC satisfaction. Further, a qualitative approach was applied to understand perceptions, practices, and policies of ANC. *Results.* There were no national guidelines for ANC in Ethiopia. Within the health system, the teaching of health professional students was given high priority, and that contributed to a lack of continuity and privacy. To the women, poor user-provider interaction was a serious concern hindering the trust in the health care providers. Further, the care provision was compromised by the inadequate laboratory facilities, unstructured health education, and lack of training of health professionals. *Conclusions.* Health system trials are needed to study the feasibility of ANC strengthening in the study area. Nationally and internationally, the leadership needs to be strengthened with supportive supervision geared towards building trust and mutual respect to protect maternal and infant health.

## 1. Introduction

We approach the countdown for the MDG5 for improved maternal health [1], and how to deliver quality antenatal care (ANC) in low-income countries becomes an important issue to address. With the MDGs, maternal health has received increasing political attention; however, little attention has been given to ANC, even though the guidelines for Focused ANC (FANC) were launched concurrently [2]. The risk of maternal death is by and large not predictable during pregnancy, and it is argued that ANC has limited effects on maternal death [3, 4]. The most important strategy for reduced maternal mortality is to ensure that women give birth by skilled attendance [5–7]. Consequently, there is an ongoing debate about the importance of ANC for maternal health. Nevertheless, pregnant women are embracing the idea of care during pregnancy; in sub-Saharan Africa, three in four pregnant women receive at least one ANC visit [1].

Generally, guidelines for FANC have not been fully implemented in low-income settings [8], and the quality of ANC is often low [9–12]. The ANC is increasingly focused on and acknowledged for providing health education, instructions on danger signs during pregnancy, and birth preparedness, as this is considered to be lifesaving and to reduce delay in seeking help [8]. The opportunities of the ANC program to counsel women in the need for skilled birth attendance have not been sufficiently investigated [13]. Thus, while the importance of a highly utilized health program is under debate, the priority of the area (hence the quality of the actual care provided) is low, and research on how to strengthen the ANC is needed.

In Ethiopia, maternal mortality rates have been among the highest in the world, although currently there are indications of a marked decline [14]. According to estimates, the maternal mortality ratio was 990 per 100,000 live births in 1990 [15] but was estimated to 676 in 2010 [16]. At national level, most recent data show that only 10% give birth under the care of a skilled provider, 51% in urban and 4% in rural settings [17]. From 2005 to 2011, the proportion attending ANC increased from 28 to 34%. Urban women were twice as likely to attend ANC as rural women were. Local studies characterise the ANC service by unclear guidelines, lack of training of providers [18, 19], and poor health system registration, but the Preventing Mother-to-Child Transmission (PMTCT) program seems to receive higher priority than the ANC program [20]. It has not been possible to identify documents providing an Ethiopian schedule for ANC. One study mentions that in 2005 FANC was not implemented [19], but in other studies [16, 18, 20] coverage of four visits is reported. FANC guidelines suggest that four visits per pregnancy will suffice, if the woman does not experience complications [2]. How ANC services are prioritised by the Ethiopian health authorities might be reflected in The National Reproductive Health Strategy 2006–2015 [21]. Overall, ANC is not central to the document, and the priority areas are social and cultural determinants of women's health, fertility and family planning, maternal and newborn health, HIV/AIDS, reproductive health of young people, and reproductive organ cancers. Thus, Ethiopia seems a relevant case for the study of how to strengthen the implementation of quality ANC for improved maternal health.

The aims of this study were to analyse if the health system priorities matched the user expectation and to assess the needs for ANC strengthening for improved maternal health in the Jimma area of Ethiopia. The specific objectives of the quantitative component were to analyse the content of ANC and to identify the predictors of low ANC satisfaction, while the specific objectives of the qualitative component were to understand perceptions, practices, and policies of ANC.

## 2. Materials and Methods

### 2.1. Study Design

The study was a needs assessment aimed at informing the development of a complex intervention study that would test the feasibility of ANC strengthening in Ethiopia. We applied the WHO guidelines for FANC as the best practice for efficient care, and the present paper provides an in-depth understanding that can guide adaption of FANC to the Jimma context. Using the terminology from the British Medical Research Council (MRC) guidance on complex interventions, this needs assessment is contributing to the initial phases of trial development, where the program theory is developed and modelled [22]. Implementation of policies for reduction of maternal mortality without involvement of local stakeholders and contextual understanding has previously been found to be ineffective [23]. Our choice of conducting an in-depth analysis of ANC in the local setting is in line with the theories for the planning of health promotion programs, where need for user-involvement and application of qualitative research is highlighted [24, 25].

### 2.2. Setting

The study was conducted in Jimma city, which has a population of 121,000, and in the surrounding urban communities of Serbo and Agaro. The ANC program of all public health facilities in the area was studied, four health centres (Jimma Town, Higher 2, Serbo, and Agaro) and one hospital (Jimma University Specialized Hospital). The hospital serves as a referral site and provides specialized care for southwestern Ethiopia, with a catchment population of about 15 million, and ANC service is provided for women with complicated as well as uncomplicated pregnancies. At the health centres, pregnant women from the nearby urban and rural communities are seen, and primary health care services (including ANC, delivery services, postpartum care, and family planning) to all population groups are provided. The registration system is poor, but an estimated 6,500 pregnant women are seen at the facilities per year. All facilities are training sites for all types of health care professionals.

### 2.3. Data Collection

Field work was conducted in the study area from August 2008 to August 2009 by SFV, with linguistic, cultural, and logistical support from AT.

The quantitative component was a questionnaire survey at the household level. All women residing in the study area who gave birth in the year preceding the interview (date of birth from April 28, 2008, to June 20, 2009) were invited to participate. We collected information about the use of and experience with health facilities during the last pregnancy, and women were asked to recall the content of care received during ANC. The questions were based on the components of care recommended in the WHO FANC model and were inspired by the Ethiopian Demographic Health Survey [26]. The questions on laboratory testing were divided into (1) urine analysis, (2) HIV test, and (3) other blood analyses (haemoglobin, blood group, Rh status, and syphilis test). The women were asked to rate how much they were satisfied with the service received during last pregnancy. User satisfaction was originally coded as follows: very satisfied, acceptable, and not satisfied, but for the regression model very satisfied and acceptable were merged. The questionnaire was in Amharic and the translation was conducted by a group of local nurses and corrected by DN and AGM independently, and the questionnaire was pilot tested. Data were collected by 23 trained female data collectors. Local guides from the* kebeles* (smallest administrative unit) helped to identify eligible women by walking from door to door. Data were double entered by trained data entry clerks.

The qualitative component included observations at ANC facilities, in-depth interviews with recent mothers and health system leaders, focus group discussions (male spouses and TBAs (Traditional Birth Attendants)), and workshops with health professionals. Observations took place regularly at all ANC facilities, adding up to 20–40 hours per site, and were focused on the infrastructure, availability of technology, types of health staff, and interaction between user and provider. Extensive field notes were taken based on these observations.

In-depth interviews were conducted with 13 recent mothers (20 days to 5 months after delivery) to understand their perceptions and experiences regarding pregnancy and utilization of ANC. We used purposeful sampling to capture the variation in ANC service delivered at different levels of care, women who followed ANC at hospital (3) or health centre level (6) or did not have any ANC at all (4). The ANC attendants were selected from the ANC archive and nonattendants from the archive of childhood immunizations. The tracing of women was difficult and time consuming due to incomplete registration at facility level and an informal address structure in the community. Apart from level of care, the sampling was therefore based on convenience. The women were interviewed in their own households by SFV and AT. Interview guidelines were developed in the local setting together with health professionals, pilot tested, and translated into the local language. The interviews were semistructured and took 25 to 45 minutes.

A focus group discussion with eight male spouses visiting Jimma Town Health Centre was conducted to gain information about their perceptions of pregnancy and to understand decision-making processes at the family level. These men attended the facility for different purposes on the day of the focus group. Further, a focus group discussion with 12 TBAs, who were recruited through a local nongovernmental organization (Family Guidance Association), was conducted to explore their roles regarding pregnancy and delivery at the community level. The discussions were conducted in the local language and facilitated by AGM. All interviews and discussions were recorded and later transcribed and translated into English.

Workshops with clinical staff members were conducted at Serbo, Jimma Town, and Higher 2 Health Centre and at the hospital (no physicians participated). The workshops consisted of two hours of group-based discussions on strengths, weaknesses, and solutions in ANC provision. The groups subsequently presented posters with their main perspectives for the rest of the group. Key informant interviews were performed with health authorities from the Town Health Bureau and managers from the obstetric department at the hospital in order to understand their perceptions of the organisation of the service as well as the regional and national priorities.

### 2.4. Data Analysis

The survey data were analysed using mixed effect logistic regression to identify predictors of being dissatisfied with the service. Health facility was analysed as the random effect parameter to adjust for the potential clustering of women using each health facility. Further adjustment was made for maternal age, socioeconomic status, and number of ANC visits. All analyses were performed in STATA 11.0 (StatCorp, Texas, USA).

The initial analysis of the qualitative interview data and focus group discussions was based on domain analysis [27]. Data were organised into categories reflecting the topics which the participants gave importance to. The categories were grouped in more superior domains, and patterns and relations between these were explored. The latter part of the analysis was inspired by hermeneutic perspectives [28]. Posters from the workshops with health professionals were analysed, and the context of the workshops and the interpersonal dynamics among the health professionals were kept in mind. Perspectives evolving from the field observations were discussed with colleagues and local health professionals for validation and contextualisation.

The quantitative and qualitative analyses were carried out with a parallel approach [29]. This indicates that the research questions of the two approaches were not initially integrated and the two approaches were designed to apply with the standards of the two disciplines separately. In the discussion of this study, however, the qualitative and quantitative findings were triangulated and the conclusions on the research question were based on whether the findings were converging, diverging, or complementary [29].

### 2.5. Ethics

Ethical permission was obtained from the Jimma University Ethical Review Committee of the College of Public Health and Medical Sciences, and permission to observe the practice at health facilities was obtained from the relevant town and zonal health bureaus as well as the hospital administration. All informants were ensured anonymity and confidentiality, and they gave their informed consent after appropriate explanation of the study objectives and content.

## 3. Results

### 3.1. Quantitative Results

Of 1392 eligible women, 1364 (98%) consented to participate. The mean (range) age was 24.5 (15–46) years (Table 1). The majority lived in Jimma (72%) and were cohabitating (92%). Almost 20% had never been to school and 42% were primiparous. During the previous pregnancy, 83% attended ANC and 67% gave birth in a health institution.

Almost 60% attended their 1st visit in the 2nd trimester (Table 2). Few women had only one visit (3%); 25% had four, whereas 44% had more than four visits. Measurements of blood pressure, weight, Tetanus Toxoid (TT) immunization, and HIV-testing were performed for more than 90% of attendants (Table 3). Abdominal examinations as well as blood tests were performed less frequently, and the numbers differed between the facilities. About 50% of women received health education. About 25% experienced discomfort due to many students or waiting for more than one hour. Overall satisfaction with the service was high (31% very satisfied and 59% acceptable), but 10% reported being* not satisfied*.

Lack of blood pressure testing, abdominal examination, HIV-testing, and TT immunization was not associated with satisfaction (Table 4). Women who did not have other laboratory tests than the HIV test were more likely to report being* not satisfied* with ANC. Moreover, health education, long wait time, poor cleanliness of the health facility, and discomfort due to many students were related to the dissatisfaction. Further, poor conduct of health professionals showed strong association with dissatisfaction.

### 3.2. Qualitative Findings

Participants in in-depth interviews were women aged 18 to 32 years, most of whom were primiparous, but also women with three and four children were interviewed. They were housewives, students, employees, and day labourers. Nine attended ANC service; two did not attend at all; and another two had contact with the service but did not classify themselves as attendants: one because she never reached the awareness that ANC is a special program for follow-up care for pregnant women.

A central aspect shared by both women and men was that women were considered vulnerable while pregnant and hence needed to be taken care of by their husbands and relatives. For some women, pregnancy added to the worry associated with poor living conditions and concerns about how to take care of their family. In general, women and men made joint decisions about health practices during pregnancy. Relatives, especially the mothers of the pregnant women, also played a significant supervising role during pregnancy. Seeking health care was seen as a way to cope with the increased vulnerability of being pregnant. Overall, the women described ANC in positive terms.

#### 3.2.1. Lack of Guidelines and Continuity of ANC Resulted in Poor Quality of Care

According to our enquiries and observations, there were no ANC guidelines in Ethiopia. The only references to guidelines were a Ministry of Health poster and a training manual for health professionals found at health centres, both based on the FANC guidelines (with four visits per pregnancy). The booking of ANC visits followed the FANC guidelines at health centres, where, in contrast, the hospital recommended monthly appointments until 28 weeks, biweekly appointments until 36 weeks, and then weekly appointments until the birth.

At all facilities, health professionals and consultation rooms were dedicated to ANC provision. At some facilities, the ANC duty would shift among all staff; at others, one person was responsible. At the health centres, the permanent staff members providing ANC were all at the nurse or midwife level; however, occasionally, the service would be provided by students, alone or under supervision. At the hospital, the ANC facility was staffed by nurses who did the measurement of blood pressure and body weight, whereas all other physical examinations and consultations were provided by medical students, who cycled through the department every 14 days, which resulted in a lack of continuity of care. The students were providing service with reference to obstetricians, but the level of supervision was low, as the obstetricians were busy attending a high number of complicated deliveries and other teaching assignments. Further, at the hospital, collaboration problems between nurses and medical students were frequent. The nurses and midwives indicated that the medical students received more attention and feedback from the senior medical staff than they did, and this made them feel less appreciated. The nurses would not have authority over medical students, so nurses refrained from taking responsibility in the presence of medical students.

During observations we noted that there was a lack of alignment of procedures in the facilities where the staff rotated or students were in charge of the ANC service. This was visible in the registration procedure, which changed from provider to provider, as well as in the placement of the equipment. During visits to the clinics, it was difficult to find a person who took the lead of providing the ANC services. In contrast, at Serbo and Higher 2 Health Centres, one person was in charge of ANC, and this created continuity and a sense of leadership. At all facilities, a standard registration book had a fixed place. Though always present, the registration procedures were faulty with no basis for reflection or adjustment of procedures. It was observed that supervision of the ANC staff and practice was minimal, which seemed to lead to apathy in the service provision. The observed lack of continuity, thorough registration procedures, and supervision were not reported a concern by the health staff during the workshops. However, they did note that preventive services were not prioritized as much as curative services. The interviews with the senior health staff indicated that these issues were part of the clinical culture and that solving them was not a priority.

At the workshops, the health professionals themselves requested training on FANC, especially in-service training that could improve their skills during their normal working hours. They felt that it was challenging to provide good-quality service due to the lack of equipment and the low standards of the physical environment.

#### 3.2.2. Lack of Laboratory Facilities and Supplies Caused Frustration

Serbo, Higher 2, and Agaro Health Centres only had laboratory facilities for HIV-testing and not for analysis of urine, haemoglobin, and syphilis and determination of blood group. Therefore, the staff referred ANC attendants to private clinics, where testing could be done based on user fees. At the hospital and Jimma Town Health Centre, the laboratory facilities were available, but, at the health centre, a minimum fee was charged: haemoglobin 3 ETB (0.17 USD), syphilis 3 ETB, blood group and Rh status 4 ETB (0.23 USD), and urine analysis 3 ETB. Iron and folic acid supplementation was not given for free at any site.

Women expected ANC service to be free, and therefore the costs of laboratory tests caused frustration. For some, tests were too expensive, and they gave up taking tests. This affected their satisfaction with the health centres: “*Unlike the hospital, the health centres are short of laboratory equipment and chemicals. If it is well equipped like the hospital it will be acceptable (female participant)*.” A woman, at her first ANC visit, was asked to take laboratory tests at a private clinic with user fees. At the second ANC visit, she was asked to take more tests at the private clinic and was refused TT immunization before she had the laboratory results. She did not have money for more laboratory services and refrained from further check-ups.

#### 3.2.3. High Priority of HIV Services Is a Contrast to the Priority of ANC

The women associated pregnancy and the need for health care with HIV, but with no other specific diseases. The health system in Jimma had previously received funding for HIV/AIDS activities, for example, training of staff. Our observations revealed that the staff members were more careful to provide good-quality service and to make thorough registrations for the PMTCT service than they were for the basic ANC service. The increased attention might be due to close follow-up and guidance by senior doctors, health authorities, and donor agencies.

#### 3.2.4. Poor Communication and Interaction between User and Provider Resulted in Broken Trust

Some women mentioned that HIV-counselling was provided, whereas the majority said that no health education was given. This inconsistency was supported by our observations, and the staff expressed a wish to improve the health education. The interactions between users and providers were dominated by the provider, and conversations were often formal and very limited. This seemed to be what both parts expected. Further, the women indicated that the service sometimes occurred without the expected content of ANC and that the staff sometimes delayed services, even though they were not busy. Poor conduct of health professionals was a recurring theme mentioned in the interviews. Both women and men reported that some health professionals had a reputation of purposely insulting the women.

A particularly important issue for the women and their partners was that ANC was not conducted in privacy, due to the presence of a high number of students. We observed that it was common to have five health providers in the room conducting ANC services for one woman. A woman put it this way: “*There was not enough privacy. Many doctors including students accompanied you. Any one up to 10 persons watched you. At that moment, it is horrible, when you exhibit an undressed body. There was no time where I was examined in privacy*.” This lack of privacy made women feel embarrassed, and several refrained from further ANC visits.

Some of the students were inexperienced and inadequately supervised. The women sometimes felt unsafe in their hands: “*I was not examined by trained health providers. Then I developed fear. It was students who gave the services. There was a woman who supervised the students. It is based on her advice that they gave you help. Since they pushed down my abdomen without care, I did not build any confidence in them (female participant)*.”

In adjacent rooms, to which the doors could not close, other services were provided. A father recommended that if many health providers had to be present, the supervision and discipline should be improved: “*If nobody enters the room during examination, it is better. If somebody enters the room during examination the mother might feel shy and will not answer the questions… the woman will be forgotten on the examination couch and the staff will start to discuss with each other. So, the door should be closed during examination*.”

Poor conduct of staff was identified as a problem by the staff themselves, but they did not reflect on the issues of lack of privacy and the negative effect on the well-being of their clients.

## 4. Discussion

### 4.1. Main Findings

The need for ANC strengthening was assessed using a mixed-method approach. There were no national guidelines for ANC in Ethiopia, and the providers in Jimma did not have support to give high priority to ANC. Within the health system, teaching of health professional students was given high priority, and that contributed to a lack of continuity and privacy for women. Poor user-provider interaction was a serious concern for the women, and contributed to a lack of trust in the providers. The interaction was deemed poor because of a combination of a poor privacy culture, a high number of students present, and a lack of dialogue and attention to the women's individual needs. Therefore, we conclude that the women and the health system have different priorities, and, from the women's point of view, the agenda of the health system compromises the care provided, and that hinders trust and mutual respect. Further, the care provision was compromised by inadequacies in leadership, supervision, skills of professionals, and routine registrations as well as a shortage of laboratory facilities. However, in the PMTCT services integrated in the ANC program, priority and funding made it possible to overcome these issues.

### 4.2. Satisfaction with Care

The measurement of satisfaction with care in the survey was relevant as an overall assessment of the women's evaluations of the care received; however, satisfaction has previously been shown to be dependent on the expectations towards care [30, 31], and, for example, primigravidas might have difficulties knowing what to expect from ANC. In this study, a relatively small proportion of women reported being not satisfied with ANC, and this might be due to underreporting dissatisfaction, as respondents tend to report favourably on questions of perceived quality of care or satisfaction [32]. By triangulating the findings of the qualitative data against those of the qualitative study, we found that the analysis of the predictors of dissatisfaction offers some guidance towards what women do not want from care. The qualitative results give a deeper understanding of why laboratory tests, privacy issues, and conduct of health staff had strong statistical association with satisfaction. Lack of health education was also significantly associated with dissatisfaction but was not a strong theme in the qualitative interviews. However, limited verbal interaction between provider and user was, and it is, interpreted as a barrier for counselling during ANC. Limited verbal interaction between user and provider has been described in other sub-Saharan settings [33, 34], and it has been ascribed in part to the fact that existing social hierarchies in the surrounding society are reflected inside the facility; poor and less-educated patients are often treated less politely and are given less attention than middle-class or wealthy patients [35, 36].

### 4.3. The Importance of User Perceptions of ANC for Place of Delivery

In this study, 67% of the women gave birth in a health facility, which is higher than the national urban average (51%), but still lower than the ANC coverage (83%). Some studies show that the use of ANC predicts the use of health institution delivery [37], whereas others do not [7, 38]. Provider attitudes have been described as barriers for use of health institution delivery [34, 39]. We hypothesize that increased attention to what women want from ANC could increase both the number of follow-up visits during pregnancy and the number of health facility deliveries. This proposition is supported by a recent multicountry study which found that the relation between the use of ANC and the place of delivery in previous studies has been underestimated and that governments and NGOs should place more importance on the role of ANC in efforts to promote skilled birth attendance [13]. Further, it could be speculated that the ability of the health professionals to improve the health of the pregnant women might increase, if the women are involved as more active partners in discussing their health status and in the screening for health problems.

### 4.4. Focus on HIV

This study revealed that both the health system and the women had high awareness of HIV, which may reflect how a vertical program can be well implemented in a less functional horizontal program. Both the high priority of HIV and the general health system falling behind have been described in other settings [40, 41]. Today the efforts to improve public health in low-income countries are partly funded by global health initiatives, and these activities lead to new inequalities in health care provision unless better integrated in the overall health system [42]. We believe that the success of the implementation of PMTCT services should be considered a model for how health system strengthening can be accomplished for improved maternal health.

### 4.5. From Policy to Practice

Although the importance of ANC for maternal health is under debate, the guidelines for FANC are based on best available evidence, ANC is implemented globally, and coverage is high. Therefore, it is surprising how poorly the FANC guidelines are implemented in low-income countries, including Ethiopia. Langley and Denis [43] suggest that quality improvement initiatives in general seem to neglect that innovations (although scientifically sound) imply a distribution of costs and benefits to several groups of stakeholders who have more or less interest in and power to support changes. Further, they argue that different stakeholders hold different values about what is good and right, and that power relations in health care systems are diffused because of the need for both professional and managerial expertise. In the present study, it was clear that quality assurance of ANC/FANC was not a shared interest; an example is that for the medical students it might be beneficial to keep the traditional model for booking of ANC visits (with many visits per woman) as they would gain more experience. They might have had relatively good chances for upholding this practice, as they seemed to be close collaborators of the senior medical doctors. Further, the collaborative challenges seen between the nurses, medical students, and senior doctors might reflect differences in values about what good care is. Clearly, the differences in the priorities of the women and the health system are an example of differences in what good care is considered to be. Finally, the diffuse leadership of ANC and hence the lack of supervision and guidelines were noted, and those were obviously due to the low priority and funding of ANC. Thus, if dedicated implementation of FANC was a goal in Ethiopia, it is clear that in the implementation process the micropolitics of power, interests, and values would be important to address.

### 4.6. Study Strengths and Limitations

The survey is based on a relatively large study population, with a very high participation rate. We cannot preclude selection bias; socioeconomically disadvantaged women, women with stillbirths, and mothers who experienced infant deaths might be underrepresented. Nevertheless, we did everything feasible to ensure inclusion of all women: local female data collectors were trained and kebele guides assisted in identifying the women. The kebele guides facilitated the acceptance of the survey in the community.

The qualitative interviews with women were conducted in their own homes because it was assumed that they would be more comfortable there. This was an important step to reduce the effects it could have when relatively powerful people from the local community together with a foreigner ask questions about the health system. In general, the participants appeared happy to meet us and to reflect on the theme introduced. At the workshops with the health professionals, it was known by all that this study was part of a needs assessment for a future intervention. Therefore, the health staff had reasons to be strategic in their expressions. Further, the health centre leaders were present, and it might have been difficult to express critical perspectives on the local leadership, including perspectives on supervision, continuity, and job responsibilities, and this is one limitation that could possibly have influenced our data. However, the extensive field stay (SFV) and the participation of local researchers (DN, AGM, and AT) in this study alleviated this risk of bias and provided a strong background for developing interview guides and for the contextualisation and interpretation of the data [44].

The findings were relatively consistent, and this consistency enhanced the validity and relevance of our conclusions [45].

### 4.7. Relevance of the Findings for Other Contexts

Data in this study stem from a single area in Ethiopia, and a discussion of transferability is relevant [45, 46]. In Jimma, the coverage of laboratory testing during ANC was higher than the national coverage; thus, the issue of poor-quality laboratory facilities seems national. From other low-income countries, it is reported that the quality of ANC is compromised by shortages of laboratory reagents and drugs, low coverage of health education, and low compliance with the FANC guidelines and also that service varies between facilities and countries [9–12, 41, 47–50]. In the present study, we found the same challenges and thus conclude that they seem to apply generally.

The focus of this study was the ANC system. However, the quality of this program depends on the health system it is nested in. On a large scale, funding and leadership are found to be cornerstones for health system strengthening in low-income countries [51], and therefore it is likely that the themes raised in this study are of particular interest for Ethiopia, but also of general interest for health system strengthening in low-income settings.

### 4.8. Implications for Practice

It seems essential that ANC services should be given increased attention and that stronger leadership should be implemented with more supervision and monitoring of the service providers. Based on our findings, one suggestion for improved continuity of care could be to avoid job rotations. Further, if many health professional students have to be present during service provision, a different culture for how to approach the women could be encouraged. In other settings, it has been suggested that improved quality of care can be achieved by in-service training and improved supportive structures at managing levels [10, 41, 47, 52].

Based on this needs assessment, an ANC strengthening intervention (the Maternity Study) is to be developed. According to the MRC guidelines for complex interventions, the intervention will be considered an exploratory trial, where the feasibility of implementing the intervention and its acceptability to providers and users will be tested [22]. The focus of the intervention should be on adaption and implementation of ANC guidelines, availability of laboratory facilities and supply, development and implementation of health education materials, in-service training of health professionals in ANC services, guidelines for ensuring privacy during consultations, and regular and supportive supervisions of ANC professionals.

## 5. Conclusions and Perspectives

The ANC program in the study area was not based on guidelines. The ANC coverage was high. Improvements are needed on guidelines development and implementation, upgrading of laboratory facilities, health education, and user-provider interaction as well as training of health professionals.

National and international decision makers need to be aware of the consequences of having one of the most utilized health care services running without guidelines and structured supervision, for example, inadequate quality of care and the loss of health professionals' motivation and user's trust in the health care system. More importance on the role of ANC in efforts to promote skilled birth attendance and maternal health is needed. However, it is clear that dedicated implementation of FANC takes strong leadership and we suggest supportive supervision of health care providers geared towards building trust and mutual respect to protect maternal and infant health.

## Figures and Tables

**Table 1:** Background characteristics of 1364 women who had given birth within the previous 12 months in the Jimma area^1^.

Age, years, mean ± SD (*n*)	24.5 ± 4.7 (1357)
Place of residence	
Jimma	71.6 (974)
Serbo	6.3 (86)
Agaro	22.1 (301)
Maternal education	
No school	19.9 (271)
Primary school	46.1 (628)
Secondary or higher school	34.0 (464)
Marital status	
Single	2.8 (38)
Widow/divorced	5.1 (69)
Cohabitating	92.1 (1254)
Parity	
Para I	42.0 (566)
Para II	26.4 (355)
Para III	14.9 (201)
Para IV+	16.7 (225)
Outcome of last pregnancy	
Singletons	98.0 (1336)
Twins and triplets	2.1 (28)
ANC attendance during last pregnancy	
Yes	83.1 (1132)
No	17.0 (231)
Place of delivery	
Health facility	67.4 (904)
Home	32.6 (437)

^1^All numbers are % (*n*) unless otherwise indicated. Numbers do not add up due to missing data.

**Table 2:** ANC utilization patterns for 1132 women who had given birth within the previous 12 months in the Jimma area^1^.

ANC facility	
Jimma Hospital	31.9 (359)
Higher 2 Health Centre	13.0 (146)
Jimma Town Health Centre	19.1 (215)
Serbo Health Centre	4.9 (55)
Agaro Health Centre	22.2 (250)
Others	8.9 (100)
Gestational age at 1st visit	
1st trimester	31.3 (336)
2nd trimester	57.8 (621)
3rd trimester	10.9 (117)
Number of visits	
1	3.1 (32)
2	7.6 (80)
3	19.7 (207)
4	25.2 (265)
5+	44.4 (466)

^1^All numbers are % (*n*). Numbers do not add up due to missing data.

**Table 3:** Reported content of care, health staff practices, and ANC surroundings (%) by facility according to 1132 ANC attendants who had given birth within the previous 12 months in the Jimma area^1^.

	Jimma Hospital (*n* = 359)	Higher 2 HC (*n* = 146)	Jimma T HC (*n* = 215)	Serbo HC (*n* = 55)	Agaro HC (*n* = 250)	Other (*n* = 100)	Total (*n* = 1125)
Content of care							
Physical examination							
Blood pressure	98.0	97.2	97.2	94.6	96.0	92.0	96.6
Weight measurement	98.3	99.3	98.1	94.6	96.0	96.0	97.5
Abdominal examination	95.8	86.3	86.5	96.4	66.8	83.0	85.2
Laboratory tests							
HIV test	96.9	95.2	95.8	76.4	92.8	82.0	93.2
Blood analyses^2^	80.6	69.7	79.3	7.4	41.5	65.7	65.3
Urine analysis	93.6	71.9	81.9	34.6	42.8	69.7	72.2
TT immunization	85.7	95.2	97.2	76.4	95.2	91.0	91.3
Health education topics							
Danger signs during pregnancy	44.1	29.9	38.9	18.5	30.8	46.5	37.2
Need for health facility delivery	62.1	65.1	54.0	30.9	38.4	58.0	53.8
HIV and PMTCT	61.1	50.7	54.0	35.3	64.0	51.1	57.2
Nutritional needs during pregnancy	64.1	71.2	59.4	47.3	39.8	66.0	58.1
Breastfeeding	59.6	58.2	58.2	47.3	46.8	59.0	55.7
ANC surroundings							
Waiting more than 1 hour preservice	43.9	27.4	16.7	1.8	16.7	11.0	25.5
Poor cleanliness of institute	5.0	0.7	0.0	3.6	13.3	1.0	4.9
Practice of health professionals							
Discomfort due to students	36.8	15.2	23.0	25.5	26.0	13.3	26.3
Poor conduct of health professionals	6.7	0.0	3.7	18.2	5.3	4.0	5.3
Nutritional practice of women							
Reduced food intake	37.1	30.8	28.5	43.6	44.8	46.9	37.5
Iron supplementation	6.8	5.6	4.2	1.9	0.8	9.1	4.8
Overall							
Not satisfied with service	9.9	2.2	6.1	25.9	17.2	6.1	10.3

^1^Numbers do not add up due to missing data, ^2^Haemoglobin, blood group, and syphilis.

**Table 4:** Predictors of being *not satisfied* with the service, as reported by 1132 ANC attendants who had given birth within the previous 12 months in the Jimma area^1^, with odds ratios (OR) and 95% confidence intervals (CI).

	Crude OR (CI)	Adjusted OR (CI)^2^
Physical examination		
No blood pressure measurement (*n* = 38)	1.6 (0.7; 4.1)	1.5 (0.5; 4.6)
No weight measurement (*n* = 30)	2.0 (0.8; 5.2)	3.1 (1.0; 9.1)
No abdominal examination (*n* = 164)	1.0 (0.6; 1.7)	1.0 (0.6; 1.9)
Laboratory tests		
No HIV test (*n* = 76)	0.6 (0.2; 1.5)	0.7 (0.3; 1.9)
No blood analysis, other (*n* = 379)	2.2 (1.4; 3.4)	2.3 (1.4; 3.8)
No urine analysis (*n* = 307)	2.0 (1.2; 3.1)	1.8 (1.1; 3.0)
No TT immunization (*n* = 98)	1.0 (0.5; 1.7)	1.2 (0.6; 2.5)
No health education on need of health institution delivery (*n* = 509)	2.8 (1.8; 4.3)	2.7 (1.7; 4.3)
Waiting more than 1 hour (*n* = 281)	4.4 (2.7; 6.9)	3.9 (2.4; 6.4)
Poor cleanliness of health institution (*n* = 55)	14.1 (7.6; 26.1)	14.1 (7.3; 27.3)
Discomfort due to students (*n* = 291)	7.9 (5.1; 12.4)	7.4 (4.6; 11.8)
Poor conduct of health staff (*n* = 60)	59.1 (28.9; 120.7)	66.0 (29.7; 146.7)

^1^The model runs with 1104 observations due to missing values on satisfaction.

^2^Adjusted for maternal age, maternal education, and number of ANC visits.
